# Vascular smooth muscle cell‐specific progerin expression in a mouse model of Hutchinson–Gilford progeria syndrome promotes arterial stiffness: Therapeutic effect of dietary nitrite

**DOI:** 10.1111/acel.12936

**Published:** 2019-03-18

**Authors:** Lara del Campo, Amanda Sánchez‐López, Mercedes Salaices, Ryan A. von Kleeck, Elba Expósito, Cristina González‐Gómez, Lorena Cussó, Gabriela Guzmán‐Martínez, Jesús Ruiz‐Cabello, Manuel Desco, Richard K. Assoian, Ana M. Briones, Vicente Andrés

**Affiliations:** ^1^ Centro Nacional de Investigaciones Cardiovasculares (CNIC) Madrid Spain; ^2^ CIBER de Enfermedades Cardiovasculares (CIBERCV) Spain; ^3^ Departamento de Farmacología y Terapéutica, Facultad de Medicina, Instituto de Investigación Hospital La Paz (IdiPaz) Universidad Autónoma de Madrid Madrid Spain; ^4^ Center for Engineering Mechanobiology and Department of Systems Pharmacology and Translational Therapeutics University of Pennsylvania Philadelphia Pennsylvania; ^5^ Departamento de Bioingeniería e Ingeniería Aeroespacial Universidad Carlos III de Madrid Madrid Spain; ^6^ Instituto de Investigación Sanitaria Gregorio Marañón Madrid Spain; ^7^ Centro de Investigación Biomédica en Red de Salud Mental (CIBERSAM) Spain; ^8^ Cardiac Imaging Unit, Cardiology Department Hospital Universitario La Paz Madrid Spain; ^9^ CIBER de Enfermedades Respiratorias (CIBERES) Spain; ^10^ Present address: CIC biomaGUNE and Ikerbasque Basque Foundation for Science San Sebastián Spain; ^11^ Present address: Universidad Complutense Madrid Madrid Spain

**Keywords:** aging, dietary nitrite, progeria, smooth muscle cells, vascular stiffness

## Abstract

Vascular stiffness is a major cause of cardiovascular disease during normal aging and in Hutchinson–Gilford progeria syndrome (HGPS), a rare genetic disorder caused by ubiquitous progerin expression. This mutant form of lamin A causes premature aging associated with cardiovascular alterations that lead to death at an average age of 14.6 years. We investigated the mechanisms underlying vessel stiffness in *Lmna^G609G/G609G^* mice with ubiquitous progerin expression, and tested the effect of treatment with nitrites. We also bred *Lmna^LCS/LCS^Tie2Cre^+/tg^*and *Lmna^LCS/LCS^SM22αCre^+/tg^* mice, which express progerin specifically in endothelial cells (ECs) and in vascular smooth muscle cells (VSMCs), respectively, to determine the specific contribution of each cell type to vascular pathology. We found vessel stiffness and inward remodeling in arteries of *Lmna^G609G/G609G^* and *Lmna^LCS/LCS^SM22αCre^+/tg^*, but not in those from *Lmna^LCS/LCS^Tie2Cre^+/tg^mice*. Structural alterations in aortas of progeroid mice were associated with decreased smooth muscle tissue content, increased collagen deposition, and decreased transverse waving of elastin layers in the media. Functional studies identified collagen (unlike elastin and the cytoskeleton) as an underlying cause of aortic stiffness in progeroid mice. Consistent with this, we found increased deposition of collagens III, IV, V, and XII in the media of progeroid aortas. Vessel stiffness and inward remodeling in progeroid mice were prevented by adding sodium nitrite in drinking water. In conclusion, *Lmna^G609G/G609G^* arteries exhibit stiffness and inward remodeling, mainly due to progerin‐induced damage to VSMCs, which causes increased deposition of medial collagen and a secondary alteration in elastin structure. Treatment with nitrites prevents vascular stiffness in progeria.

## 1 INTRODUCTION

Cardiovascular disease (CVD) is the leading cause of death and morbidity worldwide (World Health Organization, 2017). Most of the classical risk factors associated with CVD development are modifiable, for example, dyslipidemia, high blood pressure, smoking, and diabetes (D'Agostino et al., 2008). However, the most important CVD risk factor is aging, an ostensibly unmodifiable risk factor that is a defining demographic phenomenon of our times, with a high sanitary and socio‐economic impact (Population Division, 2002). It is therefore of utmost importance to gain a thorough knowledge of the mechanisms through which aging alone, independently of other modifiable cardiovascular risk factors, induces changes in the cardiovascular structure and function, in order to provide sustainable and accessible therapies to a rapidly aging population.

In addition to the characterization of risk factors epidemiologically associated and contributing to CVD development (D'Agostino et al., 2008), great advances have been made in the definition of tissue and cellular properties underlying age‐induced cardiovascular decline (Lakatta, 2003; Lakatta & Levy2003a, 2003b). Among them, vascular stiffness is attracting increasing attention due to evidence that this alteration is a key starting point for other cardiovascular complications, especially during aging (Hamczyk, del Campo, & Andrés, 2018; Lakatta & Levy, 2003a). Age‐related arterial stiffness triggers and promotes endothelial dysfunction and permeability (Huveneers, Daemen, & Hordijk, 2015), increased blood pressure, cardiac and vascular fibrosis and inflammation, inducing both vessel and cardiac overload, finally leading to atherosclerosis and heart failure (Kohn, Lampi, & Reinhart‐King, 2015; Mitchell, 2008; Wang, Monticone, & Lakatta, 2014). Furthermore, epidemiological studies indicate that vessel stiffness is a strong independent predictor of clinical cardiovascular events, especially during aging (Vlachopoulos, Aznaouridis, & Stefanadis, 2010). Therefore, targeting vessel stiffening has great potential to prevent age‐related cardiovascular disorders (Adji, O'Rourke, & Namasivayam, 2011; Boutouyrie, Laurent, & Briet, 2008; Safar, 2018).

Hutchinson–Gilford progeria syndrome (HGPS, OMIM 176670) is an ultra‐rare human genetic disease (estimated prevalence, 1 in 20 million) characterized by several signs of premature aging, including accelerated CVD (Hennekam, 2006). The disease is caused by a heterozygous de novo point mutation in the *LMNA* gene, most frequently c.1824C>T (p.G608G) (Eriksson et al., 2003; De Sandre‐Giovannoli et al., 2003). This synonymous mutation activates a cryptic splice donor site that removes 150 nucleotides from exon 11, generating a truncated form of lamin A, known as progerin, a protein that cannot undergo complete maturation and remains permanently carboxymethylated and farnesylated. As a result, progerin accumulates within the nuclear lamina and disrupts normal nuclear architecture, leading to DNA damage and many other nuclear and cell defects (Dorado & Andres, 2017; Goldman et al., 2004). The most important clinical manifestations of HGPS patients are cardiovascular complications, with patients typically dying at an average age of 14.6 years (Gordon et al., 2014). The pattern of cardiovascular deterioration is broadly similar in progeria and normal aging, although HGPS patients typically lack or are mildly affected by traditional cardiovascular risk factors (Hamczyk, del Campo & Andrés, 2018). HGPS therefore offers a unique opportunity to study mechanisms that cause age‐associated vascular dysfunction independently of other risk factors (Gerhard‐Herman et al., 2012; Hamczyk, Campo et al., 2018). Vessel stiffness is also a key player in CVD associated with HGPS, which appears very early and pervasively (Gerhard‐Herman et al., 2012; Gordon et al., 2012), and is an important cardiovascular outcome measure in HGPS clinical trials (Gordon et al., 2016, 2012). Despite the importance of vessel stiffness in the cardiovascular pathophysiology of both HGPS and normal aging, the underlying mechanisms and specific contribution of different cell types have yet to be defined.

The present study aims to investigate the mechanisms underlying vessel stiffness in HGPS by analyzing vascular structure and mechanics in mutant *Lmna^G609G/G609G^* mice, which express progerin ubiquitously and recapitulate the main clinical manifestations of human HGPS (reduced lifespan, lipodystrophy, and bone and cardiovascular abnormalities; Hamczyk, Villa‐Bellosta et al., 2018; Osorio et al., 2011; Villa‐Bellosta et al., 2013). In order to analyze the specific contribution of different cell types to the vascular pathology of progeria, we bred *Lmna^LCS/LCS^Tie2Cre^+/tg^* and *Lmna^LCS/LCS^SM22αCre^+/tg^* mice, which respectively express progerin specifically in endothelial cells (ECs) and vascular smooth muscle cells (VSMCs).

Many different therapeutic approaches for HGPS have been proposed in the last years, but clinical trials have demonstrated only very limited benefit for patients (Harhouri et al., 2018). New therapies for HGPS should be safe to permit long‐term use and should preferably target CVD, the main cause of death in HGPS (Harhouri et al., 2018). Vessel stiffness is an important determinant of CVD and is measured in HGPS clinical trials (Gordon et al., 2016, 2012; Ullrich et al., 2013). We therefore tested the effects of dietary supplementation with sodium nitrite on vascular stiffness in *Lmna^G609G/G609G^* mice, a treatment that has been shown to prevent large elastic artery stiffness during normal aging in both mouse and humans, without reported side effects (Rammos et al., 2014; Sindler et al., 2011).

## 2 RESULTS

### 2.1 *Lmna^G609G/G609G^* mice ubiquitously expressing progerin show aortic stiffness and inward remodeling that are reproduced in mice with VSMC‐specific progerin expression

**Figure 1:**
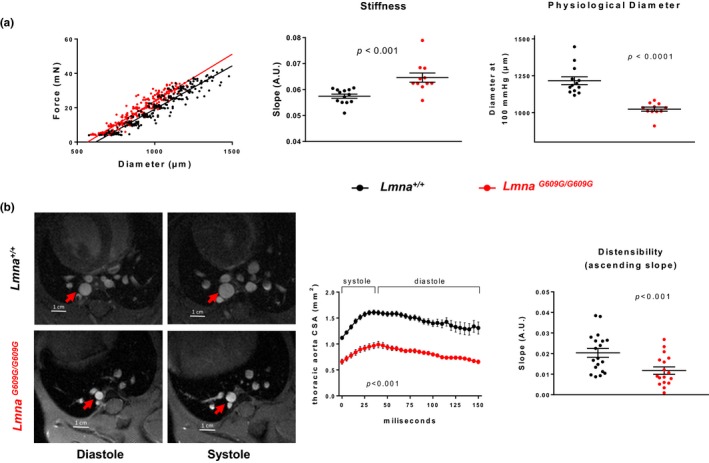
The aortas of progeroid mice exhibit arterial stiffness and inward remodeling. (a) Wire myography analysis of diameter–tension relationships, linear regression slope, and diameter estimated at 100 mmHg for aortic rings (*n* = 11 *Lmna^G609G/G609G^* mice and *n* = 13 *Lmna^+/+^* littermate controls). (b) Magnetic resonance imaging (MRI) of the thoracic aorta in *Lmna^+/+^*mice (*n* = 19) and *Lmna^G609G/G609G^* mice (*n* = 17) and quantification of aortic size in area units (mm^2^) over a complete cardiac cycle. Distensibility is expressed as the slope of the ascending part of the aortic size–time curve

The mean survival of *Lmna^G609G/G609G^*mice in our animal facility is 21.71 ± 0.82 weeks. All studies were carried out with 13‐ to 15‐week‐old male mice, which showed evident symptoms of disease but were not yet in the last stages of their lifespan. Mechanical properties of aortas were analyzed by ex vivo analysis with wire myography. These assays showed that diameter–tension relationships in aortas from *Lmna^G609G/G609G^* are left‐shifted compared to *Lmna^+/+^* controls (Figure 1a, left). Regression lines were calculated for these relations and compared. The slopes of regression lines were significantly steeper in *Lmna^G609G/G609G^* aortas, indicating increased aortic stiffness (Figure 1a, middle). Moreover, the estimated physiological diameter (diameter at 100 mmHg, Figure 1a, right) and the diameter at 0 force (Supporting Information Figure S1, left) were both decreased in aortas of *Lmna^G609G/G609G^*, indicating inward remodeling.

Since systolic and diastolic pressures are unaltered in *Lmna^G609G/G609G^* mice (Osorio et al., 2011), magnetic resonance imaging (MRI) was used to measure the stroke change in lumen area of the thoracic aorta as an in vivo measure of distensibility and stiffness (Laurent et al., 2006). We found smaller systolic and diastolic aortic diameters in *Lmna^G609G/G609G^* mice (Figure 1b, left). Moreover, the distensibility of the vessel was significantly lower in progeroid mice, as evidenced by the decreased slope of the ascending part of the cross‐sectional area–time curve (Figure 1b, right).

**Figure 2:**
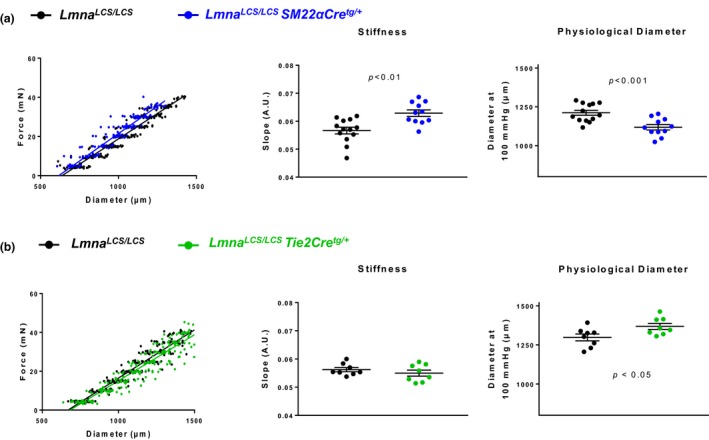
Mice with VSMC‐specific progerin expression display arterial stiffness and inward remodeling, whereas mice with EC‐specific progerin expression do not. (a, b) Wire myography analysis of diameter–tension relationships, linear regression slope, and diameter estimated at 100 mmHg for each vessel segment in aortic rings from *Lmna^LCS/LCS^SM22αCre^tg/+^*mice (*n* = 11) (a) and *Lmna^LCS/LCS^Tie2Cre^tg/+^*mice (*n* = 8) (b). Mice of both genotypes are compared with *Lmna^LCS/LCS^*littermate controls (*n* = 13 and 8, respectively)

To determine the relative contribution of VSMCs and ECs to the alterations observed in progeroid mouse aortas, we analyzed mice expressing progerin only in VSMCs (*Lmna^LCS/LCS^SM22αCre^+/tg^*) or only in ECs (*Lmna^LCS/LCS^Tie2αCre^+/tg^*). Littermate *Lmna^LCS/LCS^* mice were used as controls. Cell‐type‐specific progerin expression was confirmed by immunofluorescence on aortic sections (Supporting Information Figure S2). Wire myography revealed a steeper slope of the diameter–tension relationships and a decreased physiological diameter in the aortas of *Lmna^LCS/LCS^SM22αCre^+/tg^* mice (Figure 2a), whereas values were unaltered in aortas from *Lmna^LCS/LCS^Tie2Cre^+/tg^*mice (Figure 2b). These data indicate that VSMC‐specific progerin expression, but not EC‐specific expression, is sufficient to induce aortic stiffness and inward remodeling.

Pulse wave velocity (PWV) is the gold standard method for noninvasive measurement of arterial stiffness in humans (Laurent et al., 2006). Attempts to measure PWV in progeroid *Lmna^G609G/G609G^* mice were impeded by the presence of aortic regurgitation (data not shown), which interfered with PWV measurement. Nevertheless, we were able to measure PWV in *Lmna^LCS/LCS^SM22αCre^+/tg^* mice, since they do not develop aortic regurgitation. This analysis confirmed increased arterial stiffness in these mice relative to *Lmna^LCS/LCS^*controls (Supporting Information Figure S3), validating the MRI and wire myography results.

### 2.2 *Lmna^G609G/G609G^* mice and *Lmna^LCS/LCS^SM22αCre^+/tg^* mice show stiffness and inward remodeling in small mesenteric vessels

**Figure 3:**
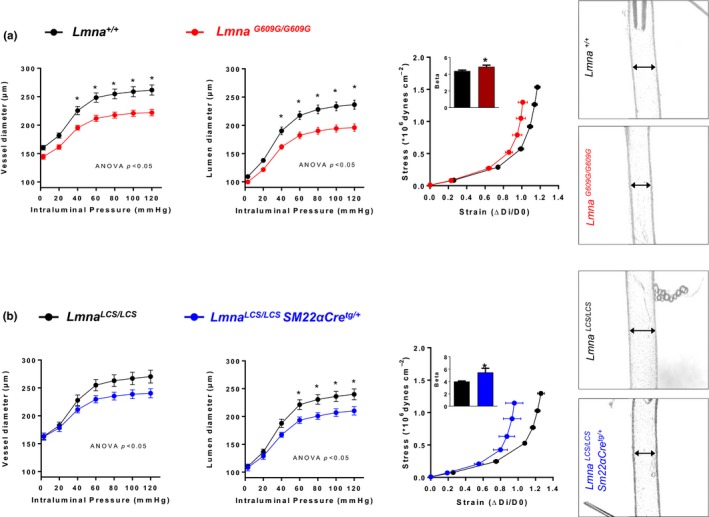
Small mesenteric vessels from ubiquitous and VSMC‐specific progeroid mice exhibit arterial stiffness and inward remodeling. Pressure–diameter curves for the vessel (outer) and lumen (inner) diameters, corresponding stress–strain curves, and representative images of the pressurized arteries at 60 mmHg. (a) Arteries from *Lmna^G609G/G609G^* mice (*n* = 10), compared with *Lmna^+/+^*littermate controls (*n* = 9). (b) Arterioles from *Lmna^LCS/LCS^SM22αCre^tg/+^*mice (*n* = 6), compared with *Lmna^LCS/LCS^* littermate controls (*n* = 6)

The structural and mechanical properties of small mesenteric arteries were studied by pressure myography, which reveals pressure–diameter properties. Vessels from *Lmna^G609G/G609G^* mice had smaller inner and outer diameters than controls, indicating inward remodeling, as well as left‐shifted stress–strain curves, indicating vessel stiffness (Figure 3a). These alterations were also observed in small mesenteric arteries from the VSMC‐specific *Lmna^LCS/LCS^SM22αCre^+/tg^*mice (Figure 3b), reinforcing the important role of VSMCs in the remodeling and stiffness of progeroid mouse arteries.

### 2.3 Collagen is an important mediator of aortic stiffness in *Lmna^G609G/G609G^* mice

**Figure 4:**
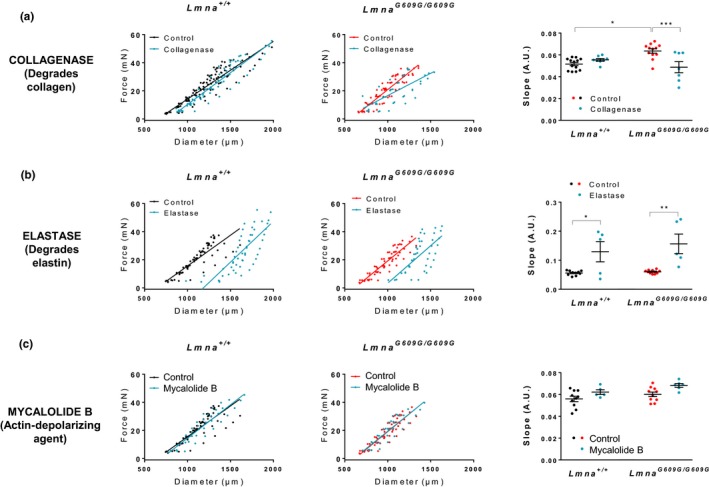
Collagen disruption prevents stiffness in aortas of *Lmna^G609G/G609G^* mice. Diameter–tension relationships and corresponding linear regression slopes in aortic rings from *Lmna^+/+^*mice (*n* = 5–10) and *Lmna^G609G/G609G^* mice (*n* = 5–10) after incubation with drugs affecting vessel structure: collagenase (collagen degradation), elastase (elastin–fiber degradation), and mycalolide B (depolymerization of F‐actin to G‐actin). Vehicle was used as control

We explored the involvement of structural components of the vessel wall that could play a role in aortic stiffness in progeroid mice, that is, collagen, elastin, and the cytoskeleton. Thus, we analyzed aortic diameter–tension relationships by wire myography in the absence and presence of specific disrupting agents: collagenase type II to degrade collagen, elastase to degrade elastin fibers, and mycalolide B to depolymerize cytoskeletal F‐actin to G‐actin. These agents can induce substantial changes in the diameter–tension relationships in control aortas, but we were particularly interested in possible differences between *Lmna^+/+^*and *Lmna^G609G/G609G^*, which could indicate a different contribution of the corresponding structure to vessel wall mechanical properties. Collagenase significantly reduced the slope of the diameter–tension relationships in *Lmna^G609G/G609G^* aortas, but had no effect on aortas from control *Lmna^+/+^* mice (Figure 4a). These results suggest that collagen is involved in the increased slope in progeroid mice and therefore in aortic stiffness. Collagenase revealed no between‐genotype differences in the estimated diameter at 100 mmHg (Supporting Information Figure S4A).

Elastase and mycalolide B had similar effects in *Lmna^+/+^*and *Lmna^G609G/G609G^*mouse aortas (Figure 4b,c), indicating that alterations in elastin and the cytoskeleton are not involved in the development of stiffness or inward remodeling in the aortas of *Lmna^G609G/G609G^* mice.

### 2.4 Aortas from *Lmna^G609G/G609G^* mice show increased collagen deposition and smooth muscle degeneration in the medial layer

**Figure 5:**
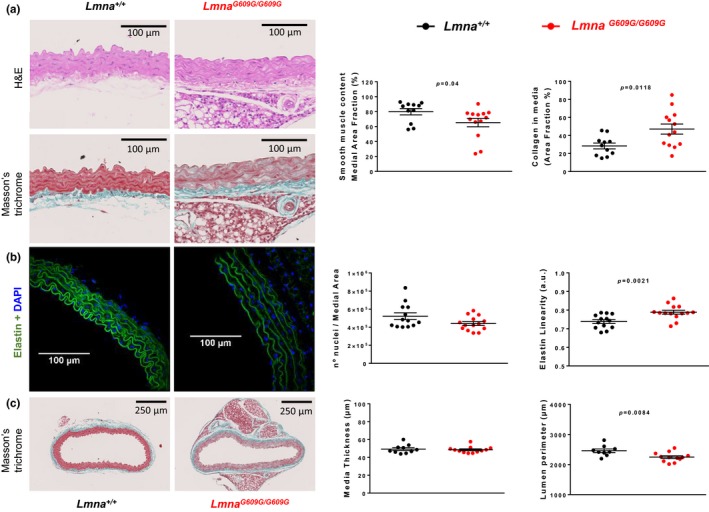
Aortic media of *Lmna^G609G/G609G^* mice shows increased collagen deposition, a decreased amount of smooth muscle tissue, and altered elastin waving. (a) Histological analysis of aortic sections from *Lmna^G609G/G609G^* mice (*n* = 13) and *Lmna^+/+^*mice (*n* = 11) stained with H&E and Masson's trichrome, showing increased collagen deposition and decreased smooth muscle area in the aortic medial layer of progeroid mice. (b) Confocal microscopy images of elastin autofluorescence and DAPI nuclear staining (*n* = 13–14), showing increased elastin wave linearity in aortic sections from *Lmna^G609G/G609G^* mice unaccompanied by significant changes in nuclear number. (c) Morphological analysis of whole aortic sections (*n* = 9–12), showing no change in medial layer thickness and a decreased lumen perimeter relative to controls, indicating inward remodeling in the *Lmna^G609G/G609G^* aorta

Histological analysis of aortic sections stained with hematoxylin–eosin (H&E) and Masson's trichrome revealed decreased smooth muscle area and increased collagen area in the medial layer of *Lmna^G609G/G609G^* aortas (Figure 5a). Fluorescent imaging of DAPI‐stained nuclei revealed no significant between‐genotype differences in cell number in the medial layer (Figure 5b), suggesting that the decrease in muscle tissue in progeroid mice is due to loss of smooth muscle mass, and not to increased cell death.

Collagen density and cross‐linking were evaluated in *Lmna^+/+^* and *Lmna^G609G/G609G^* aortas by visualizing picrosirius‐red‐stained aortic sections under polarized light. This technique detected collagen bundles only in the adventitial layer, showing no between‐genotype differences in the total amount of adventitial collagen nor in the relative amount of orange (thick) or green (thin) collagen fibers (Supporting Information Figure S5). Second‐harmonic generation microscopy was also performed to evaluate the structure of fibrous collagen. We detected collagen fibrils in the adventitial layer, but not in the media; quantification showed no differences in the structure, distribution, or amount of collagen fibers in the adventitial layer (Supporting Information Figure S6).

Morphological analysis of elastin layers, visualized as green autofluorescence, showed significant loss of elastin fiber ondulations in *Lmna^G609G/G609G^* aortas, quantified as an increase in elastin linearization (Figure 5b). We also measured the lumen perimeter and calculated media thickness in aortic histological sections. Media thickness is unaltered, while lumen perimeter is decreased in aortas from *Lmna^G609G/G609G^* mice (Figure 5c). These data agree with those obtained by wire myography, showing a decreased lumen diameter even in unloaded conditions (arteries not subjected to intraluminal force; Supporting Information Figure S1A). These results thus reinforce the idea that progeroid arteries are not only stiffer but also exhibit inward remodeling.

### 2.5 Aortas from *Lmna^G609G/G609G^* mice show increased expression of collagens III, IV, V, and XII in the medial layer

**Figure 6:**
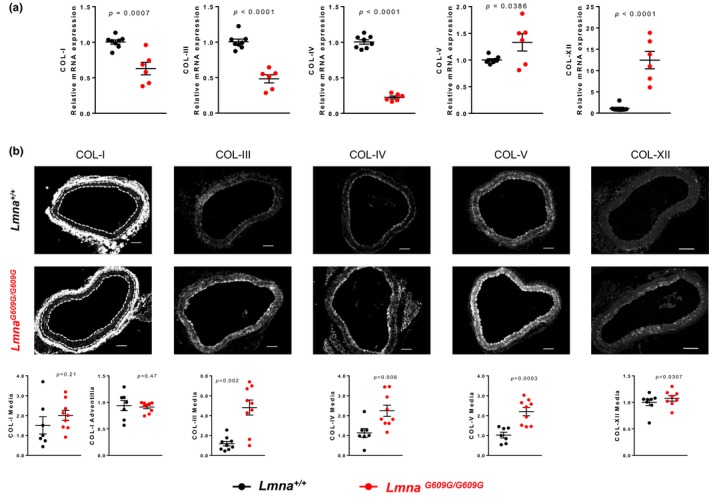
Increased deposition of collagens III, IV, V and XII in aortic media of *Lmna^G609G/G609G^* mice. Representative images of immunofluorescence staining of collagens I, III, IV, V, and XII in *Lmna^+/+^* and *Lmna^G609G/G609G^* aortic sections (*n* = 7‐10) and quantification of the normalized mean signal intensities. Media (M) layer is denoted in collagen I images between dashed lines. Scale bar 100 µm

We performed immunofluorescence experiments to examine the amount and localization of collagens in aortas from *Lmna^+/+^* and *Lmna^G609G/G609G^* *mice*(Figure 6). We focused on collagens typically present in the vessel wall that are expressed in HGPS (Stehbens, Delahunt, Shozawa, & Gilbert‐Barness, 2001), that is, collagens I, III, IV, and V. We also analyzed collagen XII since it cross‐links and organizes other collagen fibers (Chiquet, Birk, Bonnemann, & Koch, 2014). Moreover, mutations in the collagen XII gene cause Ehlers–Danlos myopathy, a very rare disease with connective tissue and vascular phenotype opposite to that seen in HGPS patients, including joint hypermobility, soft highly elastic skin, and vascular fragility (Malfait, 2018). We found that collagen I is mainly present in the adventitial layer, and no significant differences in its expression were detected in the adventitial and medial layers (Figure 6). In contrast, collagens III, IV, V, and XII were mainly expressed in the media, with significantly more deposition in the medial layer of *Lmna^G609G/G609G^* aortas (Figure 6).

### 2.6 Dietary nitrite supplementation protects against inward remodeling and stiffness in small mesenteric arteries of progeroid mice

**Figure 7:**
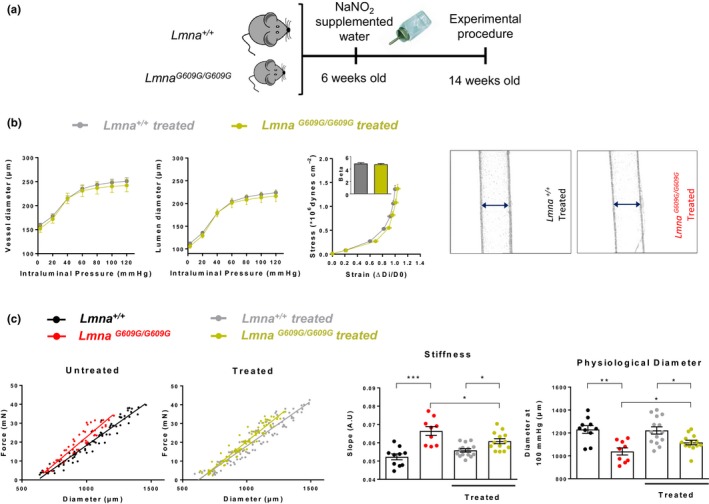
Dietary nitrite supplementation protects *Lmna^G609G/G609G^* mice against vascular stiffness and inward remodeling. (a) Study design. (b) Pressure–diameter curves for vessel (outer) and lumen (inner) diameters, corresponding stress–strain curves, and representative images of the pressurized arteries at 60 mmHg in nitrite‐treated *Lmna^G609G/G609G+/+^*(*n* = 7) and *Lmna^+/+^* (*n* = 6) mice. (c) Effect of dietary nitrite supplementation on diameter–tension relationships, slope, and physiological diameter in aortic rings from *Lmna^+/+^*mice (*n* = 10–14) and *Lmna^G609G/G609G^* mice (*n* = 9–13). Nitrites attenuate arterial stiffness, as evidenced by increases in the force–diameter slope and the physiological diameter in aortic rings of treated *Lmna^G609G/G609G^* mice

We treated control *Lmna^+/+^* and progeroid *Lmna^G609G/G609G^* mice with nitrites to explore the effect of this treatment on the observed alterations in *Lmna^G609G/G609G^* aortas (Figure 7a). Dietary nitrite supplementation at 50 mg/L prevented inward remodeling and vessel stiffness in small mesenteric arteries (Figure 7b). This dose was ineffective in aorta, but increasing the dose to 500 mg/L improved aortic vessel stiffness and inward remodeling (Figure 7c).

## 3 DISCUSSION

The present study provides insight into the mechanisms underlying vessel stiffness and vascular dysfunction in aging by studying the *Lmna^G609G/G609G^* *knock‐in* mouse model of HGPS. Like HGPS patients, these mice express progerin ubiquitously and age prematurely, and they display the main clinical manifestations of the human disease, including lipodystrophy, bone and cardiovascular abnormalities, and reduced lifespan (Hamczyk, Villa‐Bellosta et al., 2018; Osorio et al., 2011; Villa‐Bellosta et al., 2013).

We observed arterial stiffness and inward remodeling (smaller inner and outer diameters) in both the aorta and small mesenteric vessels of *Lmna^G609G/G609G^* mice. Future studies are warranted to assess whether prelamin A accumulation in progeroid *Zmpste24^−/−^*mice also causes vessel stiffening. The observed alterations in vessels of *Lmna^G609G/G609G^* mice are reproduced in arteries of *Lmna^LCS/LCS^SM22αCre^+/tg^* mice, with VSMC‐specific progerin expression, indicating that VSMCs contribute significantly to vessel stiffness in progeria. The presence of remodeling and stiffness we observed in small resistance vessels of progeroid mice should be noted as an important newly described feature in progeria, since evidence exists suggesting that associations between aortic stiffness and cardiovascular events are mediated by pathways that include microvascular damage and remodeling (Cooper et al., 2016). Vessel stiffness might therefore be a key target to threat progeria, since reversing it could improve or restore vascular and cardiac function, but also improve the function of other organs that are compromised by impaired perfusion associated with microvascular dysfunction (Chirinos, 2016; Mitchell, 2008).

Aortas from mice expressing progerin specifically in ECs (*Lmna^LCS/LCS^Tie2Cre^+/tg^* mice) showed none of these structural alterations, suggesting that ECs play no role in vascular stiffness in progeria. However, ECs appear to be critical effector cells of other progerin‐induced cardiovascular alterations, since transgenic mice overexpressing progerin only in ECs develop perivascular fibrosis in coronary arteries and interstitial myocardial fibrosis, advance to left ventricular hypertrophy associated with diastolic dysfunction, and die prematurely (Osmanagic‐Myers et al., 2018). Further studies with ubiquitous and cell‐type‐specific progerin expression mouse models are warranted to identify systemic and cell‐intrinsic mechanisms underlying primary and secondary cardiovascular anomalies in progeria. We hypothesize that early VSMC dysfunction precedes and possibly can trigger endothelial dysfunction associated with the development of atherosclerosis in HGPS consistent with our recent studies showing that VSMC‐specific progerin expression is sufficient to aggravate atherosclerosis and to cause atherosclerosis‐related premature death in *apolipoprotein E*‐null mice (Hamczyk, Villa‐Bellosta et al., 2018). However, VSMC‐specific progerin expression in atherosclerosis‐resistant mice with an intact *apolipoprotein E* gene does not affect lifespan (Hamczyk, Villa‐Bellosta et al., 2018), despite the development of vessel stiffening in aorta and small resistance vessels (Figures 2b and 3b). Further efforts are warranted to assess whether EC‐specific progerin expression is sufficient to aggravate atheroma formation in atherosclerosis‐prone mouse models (e.g., *apolipoprotein E*‐null mice and low‐density lipoprotein receptor‐null mice).

The here described new parameters to measure vessel stiffness using the wire myograph allowed us to identify the tissue and cellular mechanisms underlying structural alterations in the aortas of progeroid mice. These functional studies in which we disrupted individual vessel wall components identified collagen as an underlying cause of stiffness in the aorta of *Lmna^G609G/G609G^* mice, with no evidence for a significant contribution from elastin or the cytoskeleton. Further imaging of vessel wall components showed that arteries from progeroid animals have increased collagen deposition and decreased area of smooth muscle tissue, unaccompanied by significant decreases in cell number. VSMCs thus degenerate in the vessels of progeroid mice, and their space within the vessel wall is progressively replaced by collagen, which accounts for the increased stiffness. The change in elastin–fiber conformation to a more linear layer arrangement in *Lmna^G609G/G609G^* mice might be an indirect consequence of the alterations in collagen, and not a cause of the stiffness, since the elastase experiments showed that differences in diameter–tension relationships between control and progeroid aortas were maintained after elastin disruption. This fibrotic process caused by progerin expression in VSMCs mimics the well‐known medial degeneration and sclerosis process associated with physiological aging which promotes atherosclerosis in the long term (Sawabe, 2010). Of note, vascular stiffness in normal aging has been attributed not only to collagen accumulation, but also to elastin degradation and increased collagen cross‐linking (Kohn et al., 2015). Although our mechanistic experiment suggests collagen and not elastin as a causing agent of stiffness, we cannot rule out the involvement of increased medial collagen cross‐linking in vascular stiffness in progeria. Hence, the analysis of collagen organization and packaging by picrosirius red staining or by second‐harmonic generation imaging did not have enough resolution to detect collagen in the medial layer, but showed no between‐genotype differences in the organization and amount of collagen in the adventitia. The analysis of individual collagen types by immunofluorescence confirmed no changes in the amount of collagen in the adventitia (collagen I), and increased expression of collagens III, IV, V, and XII in the medial layer of the aorta. Further studies are required to identify the specific mechanisms by which progerin induces increased expression of different collagens, and well as to assess the relative contribution of each collagen to the stiffening of progeroid vessels.

Our findings also establish *Lmna^G609G/G609G^* *and Lmna^LCS/LCS^SM22αCre^+/tg^* mice as animal models for the study of age‐related vascular stiffness without the interference from other cardiovascular risk factors. The *Lmna^LCS/LCS^SM22αCre^+/tg^* mice will be especially useful, since they are free of other specific progeroid disease symptoms.

Nitrite supplementation of drinking water prevents vessel stiffness associated with normal aging (Rammos et al., 2014; Sindler et al., 2011), and our analysis in progeroid mice establishes that dietary nitrite protects against inward remodeling and stiffness in small mesenteric arteries and aortas in progeria. Nitrites are inorganic ions (${\text{NO}}_{2}^{-}$) usually obtained through the diet from green leafy vegetables. They have become attractive candidates for restoring physiological nitric oxide (NO) signaling in states of NO insufficiency such as aging, since they have the ability to generate NO in hypoxia or low pH conditions (in highly energy demanding tissues) through the nitrite reductase activity of a wide variety of enzymes (e.g., myoglobin or hemoglobin) (Sindler, Devan, Fleenor, & Seals, 2014). The observed protective effects of nitrites can be explained by their ability to increase NO bioavailability (Lundberg & Weitzberg, 2008; Totzeck et al., 2012), resulting in NO‐mediated protection against fibrosis (Chen et al., 2017; Kaikita et al., 2001, 2002), oxidative stress, inflammation, and mitochondrial dysfunction (Liu & Huang, 2008; Wink et al., 2001), which may protect VSMCs from progerin‐induced degeneration. Since HGPS patients die mainly of atherosclerosis and associated ischemic events, and no cure has been found yet, any therapeutic approach aiming to retard the key pathophysiological alterations that trigger their cardiovascular decline such as vessel stiffness may have a beneficial impact on the patients. Consistent with this view, clinical trials have demonstrated reduced vessel stiffness in HGPS patients treated with the farnesyltransferase inhibitor lonafarnib, which has been estimated to prolong lifespan by 1.6 years (Gordon et al., 2012, 2018; Ullrich et al., 2013). Therefore, both HGPS patients and the general aging population may benefit from the vasoprotective effects of nitrites.

## 4 EXPERIMENTAL PROCEDURES

### 4.1 Mice

*Lmna^G609G/G609G^* knock‐in mice ubiquitously express progerin (Osorio et al., 2011). Controls for these mice were *Lmna^+/^* ^+^ littermates. We crossed *Lmna^LCS/LCS^* mice (Osorio et al., 2011) with *SM22αCre* mice (The Jackson Laboratory) or with *Tie2‐Cre* mice (Kisanuki et al., 2001) to target progerin expression to VSMCs (*Lmna^LCS/LCS^SM22αCre^tg/+^*) or to ECs (*Lmna^LCS/LCS^Tie2Cre^tg/+^*). Controls used were *Lmna^LCS/LCS^Tie2Cre^+/+^* or *Lmna^LCS/LCS^SM22αCre^+/+^* littermates, respectively. All studies were carried out with 13‐ to 15‐week‐old male mice on the C57BL/6 background, and analyses were performed by researchers blinded to genotype and treatment.

Mice were maintained in the animal facility of the Centro Nacional de Investigaciones Cardiovasculares Carlos III (CNIC) under specific‐pathogen‐free conditions. All animal procedures conformed to EU Directive 2010/63EU and Recommendation 2007/526/EC regarding the protection of animals used for experimental and other scientific purposes, enforced in Spanish law under Real Decreto 1201/2005, and were approved by the local ethics committees and the Animal Protection Area of the Comunidad Autónoma de Madrid (PROEX 135/14).

### 4.2 Sample preparation

Animals were euthanized by CO_2_ inhalation. Immediately after sacrifice, the thoracic and abdominal cavities were opened. Blood samples were collected from the inferior vena cava, placed in 500 µl EDTA collecting tubes (Microvette), and maintained at 4°C for no more than 1 hr before processing to obtain plasma. Thoracic and mesenteric arteries were excised and used for different protocols.

### 4.3 Nitrite treatment

*Lmna^G609G/G609G^* and *Lmna^+/+^* mice received treatment with sodium nitrite (NaNO_2_, 50 mg/L or 500 mg/L in drinking water) over the 8 weeks before sacrifice (from 6 to 14 weeks of age) (Figure 7a) The doses used have been reported to be safe in mice, showing no evidence of toxicological or carcinogenic effects and no changes in water consumption (National Toxicology Program, 2001). Consistent with this, we observed no adverse effects or changes in water consumption in treated animals.

### 4.4 Wire myography

Thoracic aortas were placed in ice‐cold Krebs–Henseleit solution (KHS: 115 mM NaCl, 2.5 mM CaCl_2_, 4.6 mM KCl, 1.2 mM KH_2_PO_4_, 1.2 mM MgSO_4_, 25 mM NaHCO_3_, 11.1 mM glucose, and 0.01 mM EDTA) immediately after sacrifice. The vessels were gently cleaned of fat and connective tissue and cut into 2‐mm‐long segments. Wire myography was performed as previously described (del Campo & Ferrer, 2015). Aortic segments were mounted on two tungsten wires in a wire myograph system (620M, DMT) and immersed in KHS at 37°C with constant gassing (95% O_2_ and 5% CO_2_). Diameter–tension relationships were determined by artificial, stepwise stretching of the tissue, increasing its passive diameter by augmenting the distance between the wires passing through the lumen. At each step, we recorded both the force and the internal circumference of the vessel, which was transformed into vessel diameter in µm (del Campo & Ferrer, 2015). The tension experienced by the vessel wall in resisting this stretching was recorded by a force transducer connected to one of the wires, and plotted on the *y*‐axis. The estimated diameter at 100 mmHg was calculated from the diameter–tension relationship and the Laplace equation (Tension = [pressure * radius]/thickness) using the DMT normalization module (LabChart software, ADInstruments; del Campo & Ferrer, 2015). For each vessel segment, a linear regression was calculated from the diameter–tension relationship. Diameter–tension measurements were excluded when discalibration of the force transducer in a specific channel was detected (difference between the myograph unit and the software >5 mM). Diameter–tension relationships can be defined and compared with two parameters. First, the slope of the linear regression quantifies the change in tension per unit change in vessel diameter, so that a steeper slope indicates greater stiffness. Second, the extrapolated value of diameter when the force is equal to 0 represents the vessel diameter at 0 pressure (*Y*_0_ diameter), which, together with the estimated diameter at 100 mmHg (third), can indicate inward or outward remodeling.

The contribution of different vessel wall components to vessel stiffness was assessed by analyzing the diameter–tension relationships upon degradation of collagen with collagenase type II (0.2% w/v; Thermo Fisher Scientific), elastin with elastase (28 µg/µl; Sigma), or the cytoskeleton with the selective F‐actin depolymerizer mycalolide B (4 µM; Enzo Life Sciences). Aortic rings incubated with collagenase for 15 min in KHS were compared with vessels directly mounted on the wire myograph in KHS (vehicle). Incubations with elastase or mycalolide B were maintained for 3 hr at 37°C in HEPES buffer (119 mM NaCl, 20 mM HEPES, 4.6 mM KCl, 1 mM MgSO_4_·7H_2_O, 0.15 mM Na_2_HPO_4_·12 H_2_O, 0.4 mM KH_2_PO_4_, 5 mM NaHCO_3_, 1.2 mM CaCl_2_·2H_2_O, 5.5 mM glucose, pH 7.4). Diameter–tension relationships from these incubations were compared with data from control aortic rings incubated for 3 hr in HEPES at 37ºC (vehicle). Note that diameter–tension relationships and corresponding slopes for aortic rings incubated with elastase or mycalolide B share the same control data, although they are plotted separately for clarity (Figure 4).

### 4.5 Pressure myography

Structural and mechanical properties of mesenteric resistance arteries were studied with a pressure myograph (Danish Myo Tech, Model P100, J.P. Trading I/S, Aarhus, Denmark). Vessels were placed on two glass microcannulae and secured with surgical nylon suture thread. After any small branches were tied off, vessel length was adjusted so that the vessel walls were parallel without stretching. Intraluminal pressure was then raised to 120 mmHg, and the artery was unbuckled by adjusting the cannulae. The segment was then set to 45 mmHg and allowed to equilibrate for 30 min at 37°C in calcium‐free KHS (0Ca^2+^; omitting calcium and adding 1 mM EGTA), perfused intravascularly and extravascularly, and gassed with a mixture of 95% O_2_ and 5% CO_2_. Intraluminal pressure was reduced to 3 mmHg. A pressure–diameter plot was obtained by increasing intraluminal pressure in 20 mmHg steps from 3 to 120 mmHg. Internal and external diameters were continuously measured under passive conditions (D_i0Ca_, D_e0Ca_, respectively) for 3 min at each intraluminal pressure. The final value used was the mean of the measurements taken during the last 30 s, when measurements had reached a steady state.

From internal and external diameter measurements in passive conditions, the following structural and mechanical parameters were calculated:

$$\text{Wall}\;\text{thickness}\;\text{(WT)}\;=\;(D_{\text{e0Ca}}\;-\;D_{\text{i0Ca}})/2$$

$$\text{Wall:lumen}\;=\;\left(D_{\text{e0Ca}}\;-\;D_{\text{i0Ca}}\right)/2D_{\text{i0Ca}}$$

Incremental distensibility is the percentage of change in the arterial internal diameter for each mmHg change in intraluminal pressure and was calculated according to this formula:

$$\text{Incremental distensibility}\;=\;\Delta D_{\text{i0Ca}}/\left(D_{\text{i0Ca}}\;\times \;\Delta P\right)\;\times \;100$$

Circumferential wall strain (*ε*) = (*D*_i0Ca_ − *D*_00C_a)/*D*_00Ca_, where *D*_00Ca_ is the internal diameter at 3 mmHg, and *D*_i0Ca_ is the observed internal diameter for a given intravascular pressure, both measured in 0Ca^2+^ medium.

Circumferential wall stress (*σ*) = (*P* × *D*_i0Ca_)/(2WT), where *P* is the intraluminal pressure (1 mmHg = 133.4 × 10^3^ dynes·cm^‐2^), and WT is wall thickness at each intraluminal pressure in 0Ca^2+^‐KHS.

Arterial stiffness independent of geometry is determined by Young's elastic modulus (*E* = stress/strain) (Briones et al., 2009; Schjorring, Carlsson, & Simonsen, 2015). The stress–strain relationship is nonlinear; therefore, it is more appropriate to obtain a tangential or incremental elastic modulus (*E*_inc_) by determining the slope of the stress–strain curve (*E*_inc_ = *δσ*/*δε*). *E*_inc_ was obtained by fitting the stress–strain data from each animal to an exponential curve using the equation $\sigma \;=\;\sigma_{\text{orig}}{\text{e}}^{\beta \varepsilon }$, where *σ*_orig_ is the stress at the original diameter (diameter at 3 mmHg).

Taking derivatives from the equation, we determine that *E*_inc_ = *βσ*. For a given *σ*‐value, *E*_inc_ is directly proportional to *β*. An increase in *β* implies an increase in *E*_inc_, which signifies an increase in stiffness.

### 4.6 Histological analysis

Thoracic aorta segments were fixed in 4% paraformaldehyde. Following dehydration in an ascending ethanol series, samples were embedded in paraffin, cut into 5‐µm sections, and stained with H&E, Masson's trichrome, or picrosirius red. H&E staining was performed to check primary tissue appearance. Samples stained with H&E and Masson's trichrome were imaged with OPT Scanner 3001 (OPT, Bioptonics Microscopy). Medial thickness and lumen perimeter length were measured in Masson's trichrome‐stained specimens. Collagen and VSMC content were measured in the medial layer as the green or red area, respectively, in Masson's trichrome‐stained specimens (Fiji software; ImageJ 1.50e x64) and expressed as a percentage of the medial area.

Picrosirius‐red‐stained slices were imaged under a Nikon Eclipse 90i microscope both in bright field and under polarized light in order to visualize collagen bundles with different thickness and packaging (Rittie, 2017). Under polarized light, denser collagen bundles are seen as yellow‐orange, whereas the less dense or thinner collagen bundles appear green (Lattouf et al., 2014). Under polarized light, we observed green or yellow‐orange collagen bundles only in the adventitial layer, and quantified them with ImageJ software.

To analyze elastin lamellae and nuclei, 5‐µm aortic sections were stained with DAPI (1:500; Invitrogen), mounted in Eukitt^®^ mounting medium (Sigma‐Aldrich), and imaged with a confocal microscope (Leica SP5 DMI 6000B) taking advantage of the intrinsic autofluorescence of elastin fibers. The semiautomatic image processing and quantification of elastin “rectilinearity” were developed as a toolbox in Fiji (ImageJ 1.50e 64‐bit for Windows). Rectilinearity measures the length and waviness of each elastin fiber. Thus, a region of interest with elastin fibers and a threshold to segment them are selected. A skeletonization of the segmented objects (Lee, Kashyap, & Chu, 1994), after removing small connected components, extracts the center lines of each lamellae independently of their intensity or thickness; these are later analyzed to measure the length (“fiber length”) of each resulting branch and the corresponding Euclidean distance between starting and final points (“fiber distance”; Arganda‐Carreras, Fernandez‐Gonzalez, Munoz‐Barrutia, & Ortiz‐De‐Solorzano, 2010). The final output is the ratio between the accumulative sum of fiber distance and fiber length for all branches. A first preprocessing step was applied to remove noise from the original image by applying a median filtering followed by background subtraction using the “rolling ball” algorithm (Sternberg, 1983).

### 4.7 In vivo magnetic resonance imaging

For the MRI acquisition, mice were anesthetized with 2% isoflurane and a 1.8 L/min oxygen flow. Ophthalmic gel was placed on the eyes to prevent drying. Thoracic aorta cine MR studies were acquired using a 7‐T Agilent/Varian scanner (Agilent, Santa Clara, CA, USA) equipped with a DD2 console, an actively shielded 115/60 gradient set, and a microstrip helmet coil used for both RF transmission and reception. MRI sequences were based on an ECG‐triggered fast gradient echo cine sequence with the following imaging parameters: 181.82/2.07 ms (minimum repetition time TR, minimum echo time TE); field of view, 3.0 cm^2^; acquisition matrix, 256 x 256; flip angle, 40º; 8 averages; 20 cardiac phases; 2 slices; slice thickness, 0.2‐0.4 mm; and slice gap, 0.6 mm.

To determine the descending aorta cross section, we acquired two transverse images perpendicular to the long axis of the aorta and spinal cord, located slightly above the heart apex level, set on three orthogonal planes (transverse, coronal, and sagittal) used to localize the thoracic aorta, spinal cord, lungs, and heart.

For image quantification, aortic lumen area was measured on the cine MR images by manual segmentation by a trained operator, using the freely available Segment software v1.9 R3819 (http://segment.heiberg.se) (Heiberg et al., 2010). In some images, the area could not be determined, and therefore, we used the upper section data, with data from the lower section used to fill in missing values when necessary. Since the time sampling was different for each animal (depending on heart rate), a time linear interpolation was applied to all curves in order to obtain equivalent points throughout the cardiac cycle. For curve plotting purposes, the mean and standard deviation at each time point was determined for both animal groups. All time curves were cut at 150 ms, as the different cycle duration for each animal prevented a proper time alignment beyond that point. From the original area raw data in mm^2^, the following derived parameters were calculated: distensibility or initial ascending slope (estimated by linear fitting over the first 20 ms at systole); incremental area (measured as area difference from the first point); strain (measured as the aortic radius increase divided by initial radius). Total strain was defined as the integral of the strain curve.

### 4.8 Immunofluorescence staining

Thoracic aorta segments were fixed for 24 hr in 4% paraformaldehyde, embedded in paraffin, cut into 5‐μm sections, deparaffinized, and rehydrated. Sections were antigen‐unmasked, permeabilized, and incubated with blocking solution. Primary antibodies were incubated overnight at 4ºC in blocking buffer. Sections were washed and incubated with appropriate secondary antibodies for 1‐2 hr at room temperature and with DAPI/Hoechst, mounted with Fluoromount™ (Sigma) or Thermo Fisher SlowFade Gold antifade reagent (S36936), and visualized in a confocal microscope (Leica SP5 DMI 6000B for collagen XII and Lamin A; Nikon Eclipse 80i for collagens I, III, IV, V).

For lamin A staining, antigen retrieval was performed with 0.37 g/L EDTA buffer (pH 8) for 30 min, permeabilization with 0.5% Triton X‐100 for 10 min, and blocking with 5% BSA. For collagen XII staining, antigen retrieval was performed with citrate buffer (pH 6) for 20 min in the microwave, and blocking and permeabilization with 0.3% Triton X‐100, 5% normal goat serum, and 5% BSA in PBS. For the rest of collagens, permeabilization was not necessary and antigen retrieval was performed with Antigen Unmasking Solution (H3300, Vector Labs). Blocking was performed with 2% BSA solution in PBS for 15 min.

Antibodies used were as follows: antilamin A H‐102 antibody (1:100; Santa Cruz Biotechnology); anticollagen XII antibody (1:100, courtesy of Manuel Koch, Germany); COL‐I (Southern Biotech 1310‐01, 1:400); COL‐III (Proteintech 22734‐1‐AP, 1:250 in PBS); COL‐IV (Abcam ab6586, 1:250); COL‐V (Abcam ab7046, 1:250).

Collagen signal was quantified using ImageJ. Mean fluorescence intensity was obtained for the selected area of the medial layer (and the adventitial layer, in the case of Collagen I). Intensity values were then normalized to the median of the controls. For collagen XII, due to background, maximum entropy threshold was used to select collagen XII‐positive pixels, and integrated density was obtained and normalized to the median of the control mice.

### 4.9 Second‐harmonic generation microscopy

Second‐harmonic generation microscopy was used to visualize the three dimensional organization of collagen throughout the arotic wall and to examine structural differences. H&E‐stained thoracic aorta sections were used for the quantification of backward second‐harmonic generation signal from collagen fibrils, which was obtained by multiphoton excitation at 860 nm using a tunable femtosecond pulsed laser (MaiTai DeepSee, Spectra‐Physics) coupled to a Zeiss LSM780 upright system and a water‐dipping Plan‐Apochromat 10x/NA 0,45 objective (Carl Zeiss Jena GmbH). Quantification was performed by ImageJ to obtain the amount and density of the collagen signal, as well as kurtosis and skewness parameters. Kurtosis measures the degree to which a distribution is more or less peaked than a normal distribution, and skewness measures the degree of asymmetry of a distribution (Mostaco‐Guidolin et al., 2013). Since second‐harmonic generation imaging detected collagen bundles only in the adventitia, the results obtained from the quantification of these images refer only to the adventitial layer.

### 4.10 Pulse wave velocity

PWV is the velocity at which the arterial wave propagates through the circulatory system. Aortic‐femoral PWV was measured by Doppler ultrasound using the transient time (TT) system (Laurent et al., 2006). Animals were anesthetized (1.5% isoflurane in oxygen) and maintained in supine position on a temperature‐controlled surface to maintain body temperature at 37ºC with continuous electrocardiographic (EKG) recording. The pulse wave was recorded using a pulsed wave Doppler ultrasound in a VEVO 2100 system (VisualSonic). The ascending aorta was located in 2D mode, and the wave Doppler flow was then recorded simultaneously with EKG. The process was repeated on the femoral artery at the level of the thigh. The time from the QRS R wave to the foot of the pulse waveform was measured in both the ascending aorta and femoral artery. The TT is the difference between these times measured at the two measurement points. PWV is calculated as the distance between the two measurement points divided by the pulse wave TT.

### 4.11 Statistical analysis

Results are represented as mean ± standard error of the media (*SEM*). The Student *t* test was used to compare univariable data between the two groups. Single‐variable comparisons between more than two groups were performed with one‐way ANOVA followed by the Sidak multiple comparisons tests. Outliers identified using the maximum normalized residual test (Grubbs’ test) were excluded. Diameter–tension relationships were analyzed by calculating the linear regression lines and their corresponding slopes and diameter at force 0 (*Y*_0_), which were then compared among groups using the Student *t* test or one‐way ANOVA. Diameter–pressure curves were compared with two‐way ANOVA followed by the Sidak multiple comparisons tests. Stress–strain curves were analyzed by extracting the *β* value for each curve (see Section 2.6, Pressure myography), which we compared among groups using the Student *t* test. Aortic cross‐sectional area curves obtained by MRI and collagen immunofluorescence measurements were compared by the Mann–Whitney *U* test. Results were considered statistically significant at *p*‐values <0.05. Statistical analysis was performed with GraphPad Prism 7 software.

## CONFLICT OF INTEREST

None declared.

## AUTHOR’S CONTRIBUTION

L.d.C and V.A. conceived and designed the study, and wrote the manuscript; L.d.C. performed experiments, and analyzed and interpreted results; A.S.‐L. was involved in the design of the study, performed experiments, analyzed and interpreted results, and participated in manuscript writing; A.M.B. carried out, analyzed, and interpreted pressure myography experiments; R.A.K and R.K.A. carried out, analyzed, and interpreted collagen immunofluorescence studies; C.G.‐G. and E.E. provided technical support in the laboratory experiments; L.C., J.R.‐C., and M.D. analyzed and interpreted MRI data; G.G.‐M. revised and interpreted PWV experiments; M.S. revised and interpreted pressure myography experiments; V.A. interpreted results and supervised the study. All authors discussed the results and made a critical revision of the manuscript.
