# Supplementary material for: Vascular smooth muscle cell‐specific progerin expression in a mouse model of Hutchinson–Gilford progeria syndrome promotes arterial stiffness: Therapeutic effect of dietary nitrite
Source: Aging Cell. 2019 Mar 18;18(3):e12936. doi: 10.1111/acel.12936 (PMC6516150; doi:10.1111/acel.12936)
Supplement: Supplementary file 1 [file ACEL-18-e12936-s001.pdf]

Supporting information

Vascular smooth muscle cell-specific progerin expression in a mouse model of Hutchinson-Gilford progeria syndrome promotes arterial stiffness: Therapeutic effect of dietary nitrite

Running title: VSMCs and arterial stiffness in progeria

Lara del Campo, PhD[1,2,*], Amanda Sánchez-López, MSc[1,2,*], Mercedes Salaices, PhD [2,3], Ryan A. von Kleeck [9],Elba Expósito[1,2], Cristina González-Gómez[1,2], Lorena Cussó, PhD[1,4,5,6], Gabriela Guzmán-Martínez, MD, PhD[1,7], Jesús Ruiz-Cabello, PhD[1,8,**], Manuel Desco, PhD[1,4,5,6], Richard K. Assoian [9], Ana M. Briones, PhD [2,3] and Vicente Andrés, PhD [1,2,‡]

[1] Centro Nacional de Investigaciones Cardiovasculares (CNIC), Madrid, Spain

[2] CIBER de Enfermedades Cardiovasculares (CIBERCV), Spain

[3] Departamento de Farmacología y Terapéutica, Facultad de Medicina, Universidad Autónoma de Madrid, Instituto de Investigación Hospital La Paz (IdiPaz), Madrid, Spain

[4] Departamento de Bioingeniería e Ingeniería Aeroespacial, Universidad Carlos III de Madrid, Madrid, Spain

[5] Instituto de Investigación Sanitaria Gregorio Marañón, Madrid, Spain

[6] Centro de Investigación Biomédica en Red de Salud Mental (CIBERSAM), Spain

[7] Cardiac Imaging Unit, Cardiology Department, Hospital Universitario La Paz, IdiPaz, 28046 Madrid, Spain.

[8] CIBER de Enfermedades Respiratorias (CIBERES), Spain

[9] Center for Engineering Mechanobiology and Department of Systems Pharmacology and Translational Therapeutics, University of Pennsylvania, Philadelphia, PA 19104.

* These authors contributed equally to this study

** Current affiliation and address: CIC biomaGUNE and Ikerbasque- Basque Foundation for Science, Paseo Miramón 182, 20014 San Sebastián (Spain), and Universidad Complutense Madrid.

‡ Corresponding author:

Vicente Andrés
CNIC, Melchor Fernández Almagro 3, 28029 Madrid (Spain)
Phone: +34-91 453 12 00 (Ext. 1502)
E-mail: vandres@cnic.es

KEY WORDS: aging, progeria, vascular stiffness, smooth muscle cells, dietary nitrite

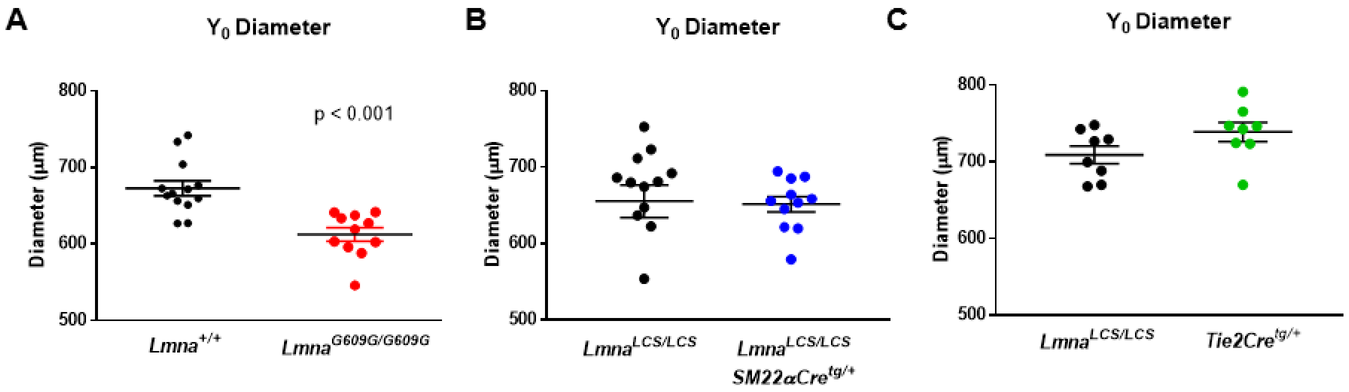

Supplementary Figure S1. Aortic diameter at unloaded conditions (no distension). Y_0 diameter was obtained from diameter-force relations by wire myography in aortic rings from *Lmna*$^{G609G/G609G}$ mice (A), *Lmna*$^{LCS/LCS}$*SM22αCre*$^{tg/+}$ (n=11) (B) and *Lmna*$^{LCS/LCS}$*Tie2Cre*$^{tg/+}$ (n=8) (C) mice, which are compared with corresponding littermate controls *Lmna*$^{+/+}$ and *Lmna*$^{LCS/LCS}$ respectively (n=13 and 8, respectively).

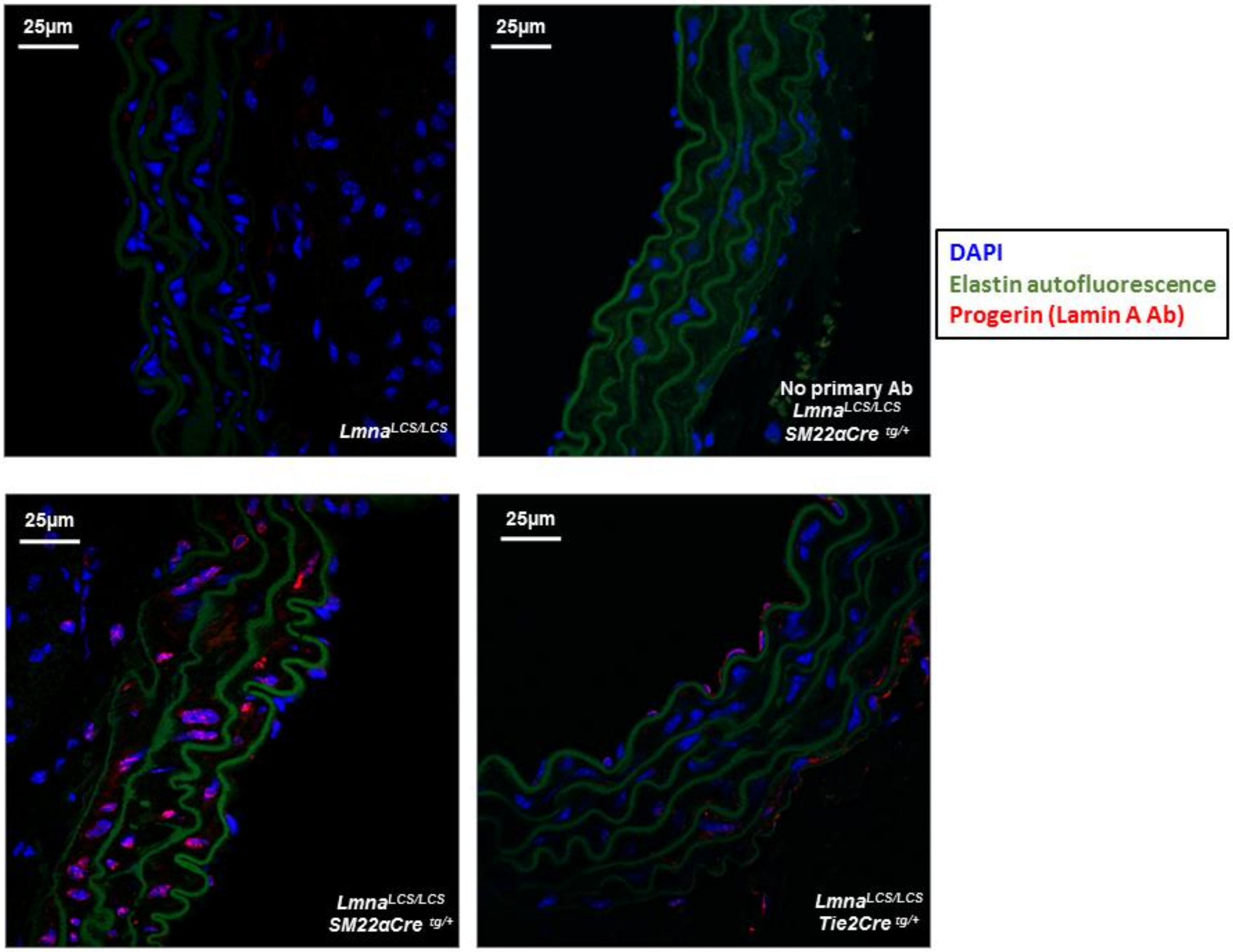

Supplementary Figure S2. Cell-specific progerin expression in aorta from *Lmna*$^{LCS/LCS}$*SM22αCre* $^{tg/+}$ and *Lmna*$^{LCS/LCS}$*Tie2Cre* $^{tg/+}$ mice. Immunofluorescence imaging of thoracic aortic sections from *Lmna*$^{LCS/LCS}$, *Lmna*$^{LCS/LCS}$*SM22αCre* $^{tg/+}$ and *Lmna*$^{LCS/LCS}$*Tie2Cre* $^{tg/+}$ mice stained with DAPI for nuclei and an anti-Lamin A antibody that recognizes progerin but not Lamin C. Elastin is also visualized due to its green autofluorescence. Negative control without primary antibody (No primary Ab) was performed in an aortic section of *Lmna*$^{LCS/LCS}$*SM22αCre*$^{tg/+}$ mice incubated with vehicle instead of primary Anti-Lamin A antibody.

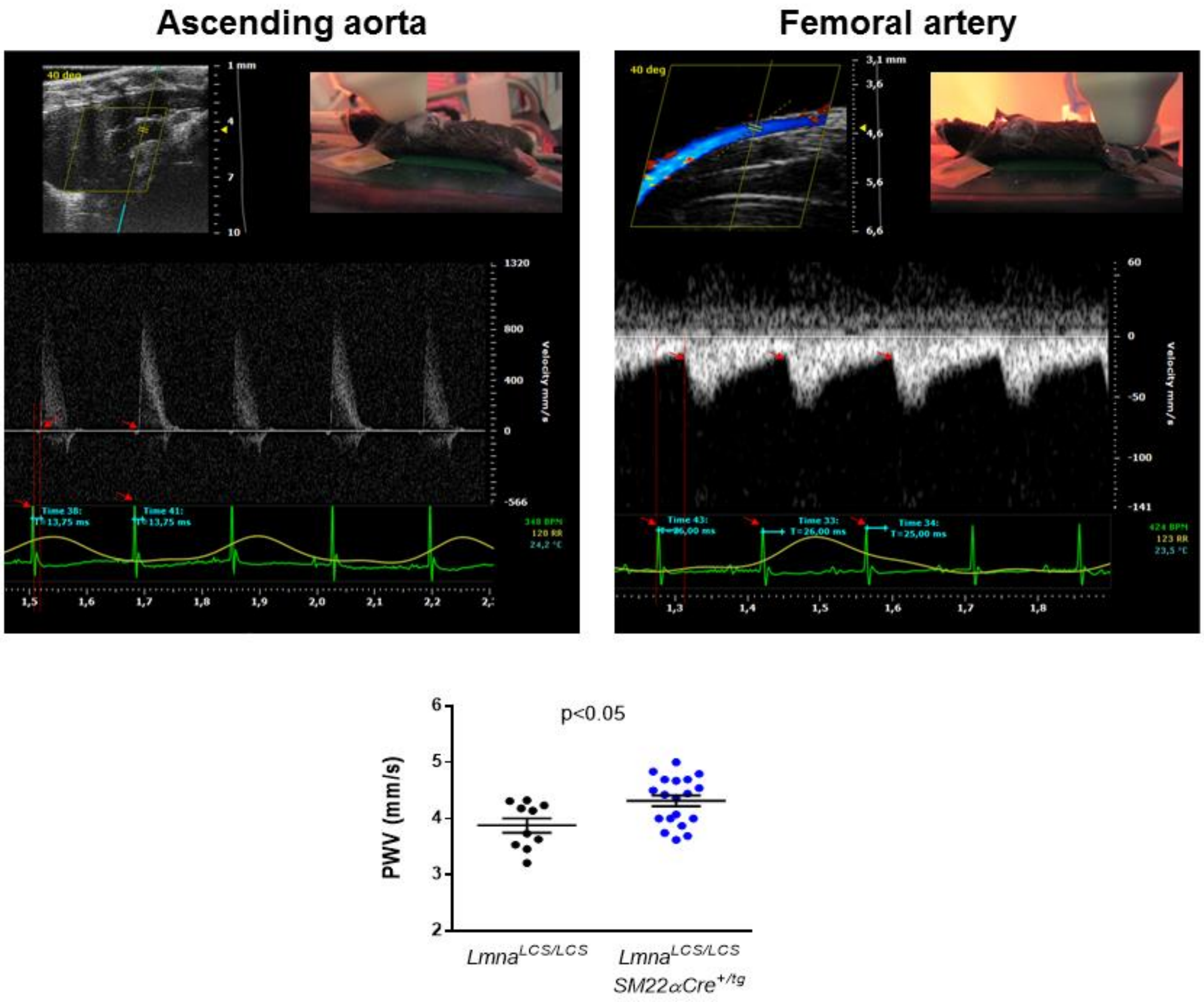

Supplementary Figure S3. Aortic-femoral aorta stiffness measured by pulse wave velocity (PWV) in *Lmna*$^{LCS/LCS}$ and *Lmna*$^{LCS/LCS}$ *SM22αCre*$^{tg/+}$ mice. PWV was measured in control *Lmna*$^{LCS/LCS}$ mice and in *Lmna*$^{LCS/LCS}$ *SM22αCre*$^{tg/+}$ mice with VSMC-specific progerin expression (PWV could not be measured in *Lmna*$^{G609G/G609G}$ mice because they presented aortic regurgitation).Stiffness of the artery is directly proportional to the velocity of the pulse wave. The velocity of the pulse wave is calculated by measuring the time from the R wave of QRS to the foot of the pulse waveform in both the aorta and the femoral arteries. The distance from the aortic arch to the femoral artery is divided by the transit time (TT) to get the velocity of the pulse wave.

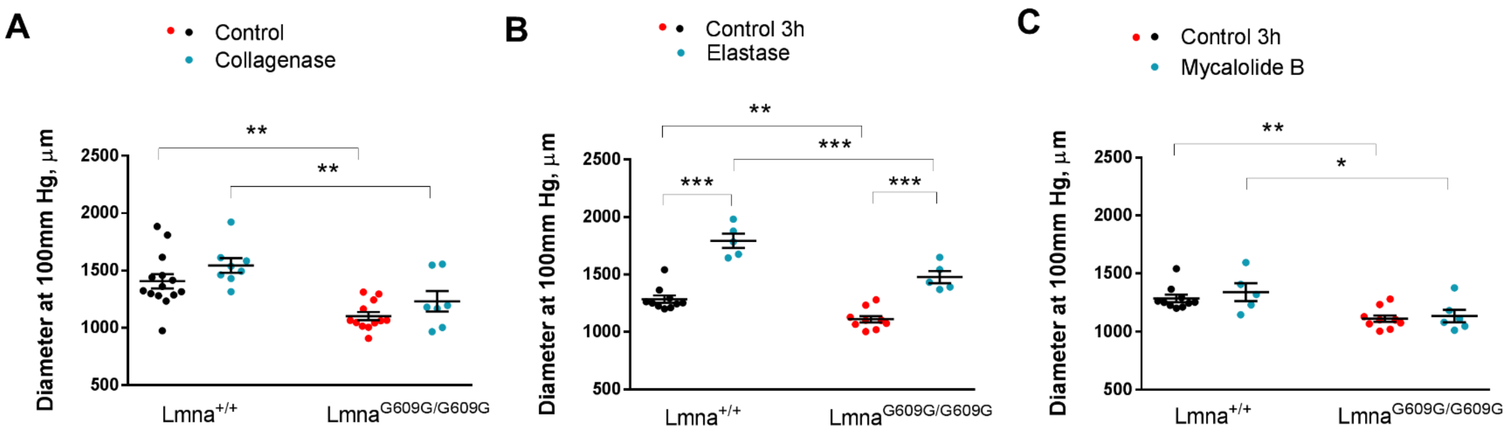

Supplementary figure S4. Effect of collagenase, elastase, and mycalolide B on physiological diameter. Diameter was estimated at 100 mmHg obtained from diameter-force relations by wire myography in aortic rings from *Lmna*$^{+/+}$ and *Lmna*$^{G609G/G609G}$ mice. Collagenase (A) and elastase (B) degrade collagen and elastin respectively, and mycalolide B (C) depolymerizes F actin to G actin, thus disrupting the cytosqueleton.

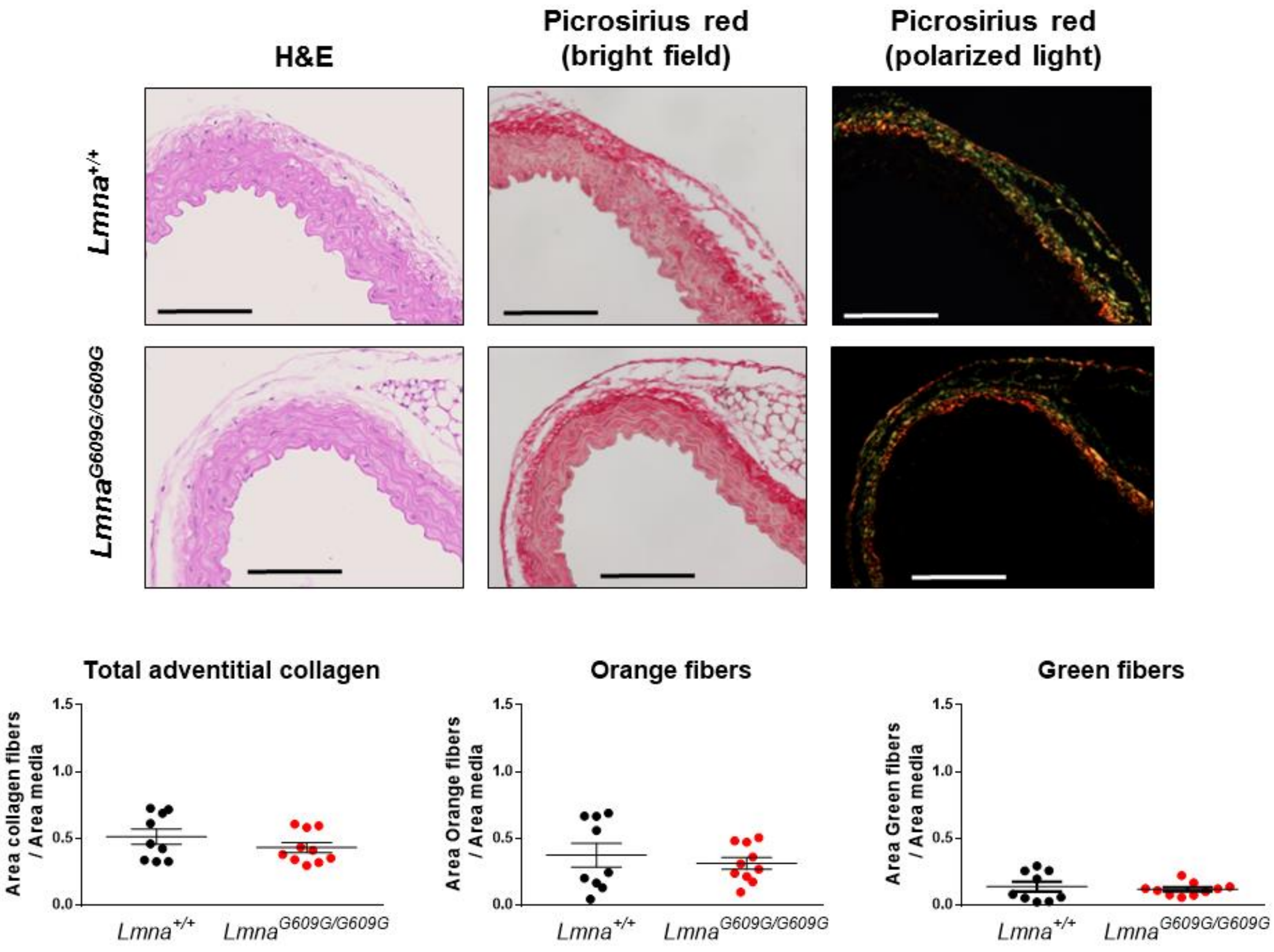

Supplementary Figure S5. Adventitial collagen is not altered in progeroid mice. Histologic analysis of aorta from *Lmna*$^{G609G/G609G}$ mice (n=9) and *Lmna*$^{+/+}$ (n=10) mice stained with Haematoxylin/Eosin (H&E), and Picrosirius Red. Picrosirius red-stained specimens were visualized both under bright field or polarized light, the latter of which allows visualizing collagen bundles, being the green ones the less compacted and the orange ones the more dense, compacted or crosslinked collagen fibers. Under polarized light, collagen bundles were only detected in the adventitia. Quantification of this adventitial collagen bundles shows no changes in the total amount of detected collagen nor in the relative amount of the thicker (Orange) of the thinner (Green) fibers between *Lmna*$^{G609G/G609G}$ and *Lmna*$^{+/+}$ aortic sections. Scale bar 100µm.

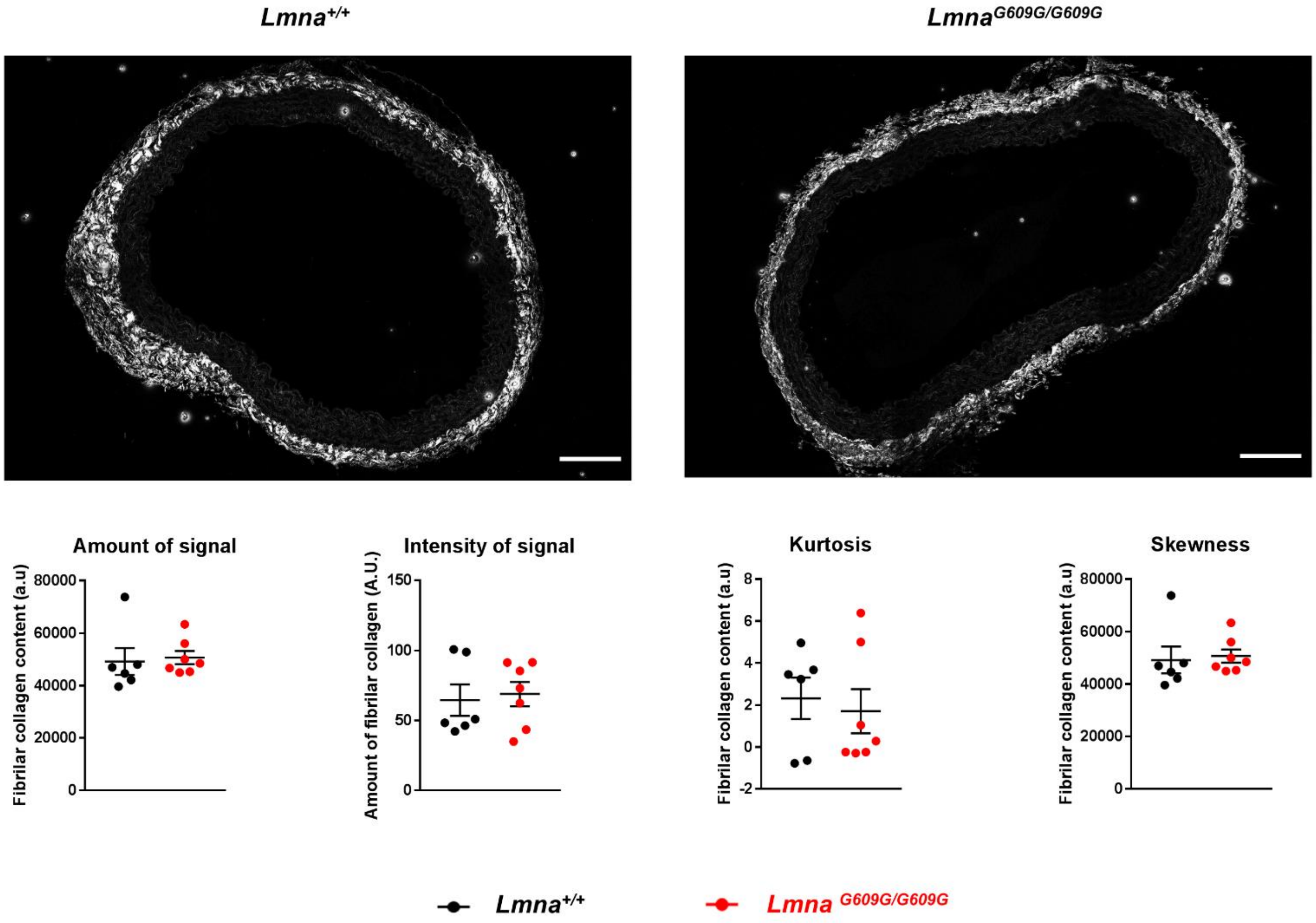

Supplementary Figure S6. Second Harmonic Generation (SHG) imaging in the adventitia layer in thoracic aortas from *Lmna*$^{+/+}$ and *Lmna*$^{G609G/G609G}$ mice. SHG imaging of thoracic aortas from *Lmna*$^{G609G/G609G}$ (n=7) and *Lmna*$^{+/+}$ mice (n=6) detected collagen bundles only in the adventitial layer. Quantification showed no differences in the collagen amount, organization or distribution in the adventitia layer. Scale bar 100µm.

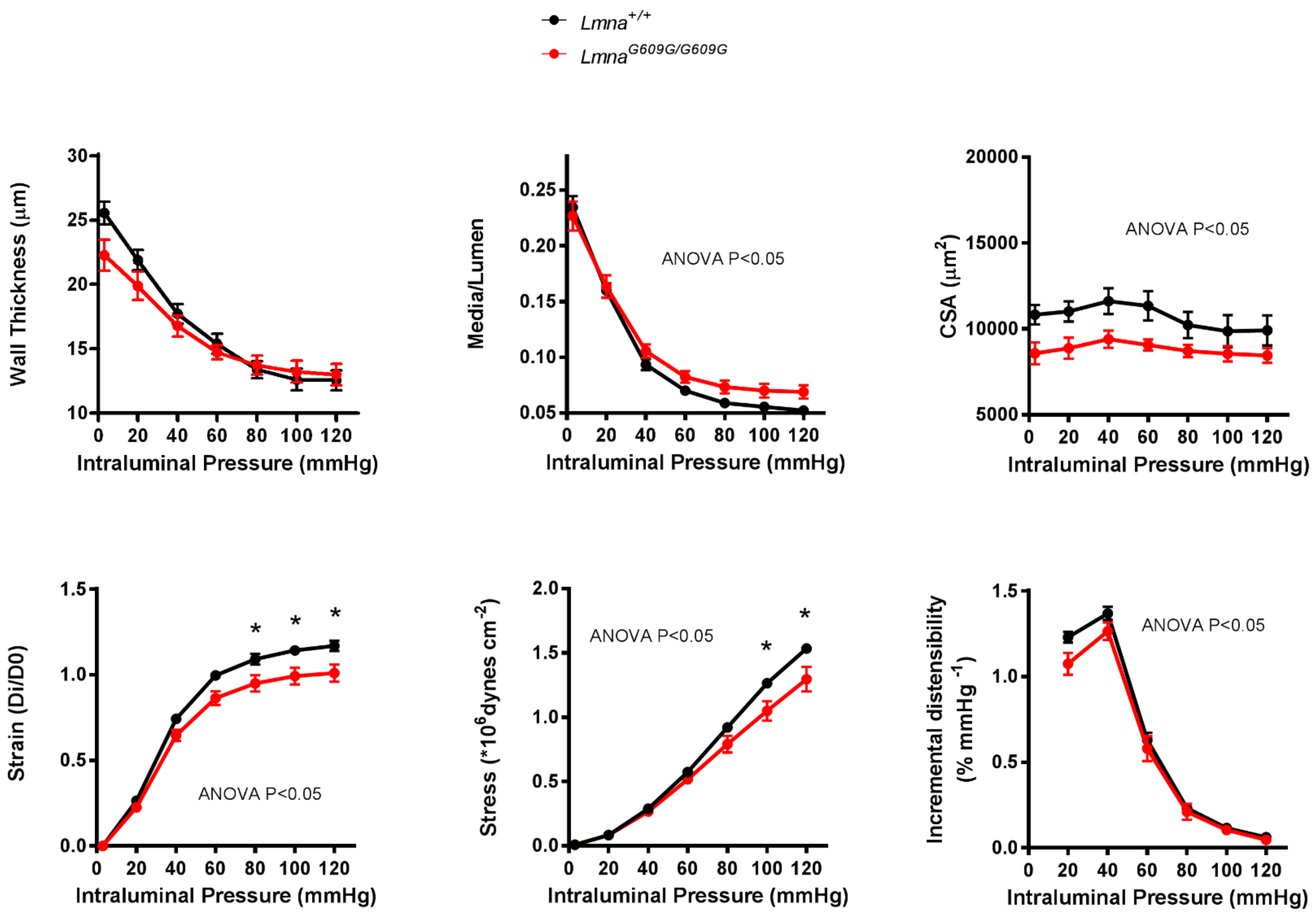

Supplementary Figure S7. Comparison of different parameters obtained from pressure-diameter curves with the pressure myograph in small mesenteric arteries from *Lmna*$^{+/+}$ and *Lmna*$^{G609G/G609G}$ mice. Wall thickness and Media/Lumen ratio are maintained, while cross sectional area of the vessel wall is decreased in small mesenteric arteries from *Lmna*$^{G609G/G609G}$ mice (n=9) compared with control *Lmna*$^{+/+}$ mice (n=9).
